# A Software and Hardware Cooperation Method for Full Nyquist Rate Transmission Symbol Synchronization at E-Band Wireless Communication

**DOI:** 10.3390/s22228924

**Published:** 2022-11-18

**Authors:** Fei Wang, Zhiqun Cheng, Hang Li, Dan Zhu

**Affiliations:** 1 School of Electronics and Information, Hangzhou Dianzi University, Hangzhou 310018, China; 2 Hangzhou Dianzi University Information Engineering College, Hangzhou 310018, China

**Keywords:** full Nyquist rate transmission, symbol synchronization, phase adjustable clock, initial phase acquisition, phase tracking, E-band

## Abstract

Compared with the conventional pulse-shaping transmission system, the full Nyquist rate transmission system with large bandwidth is sensitive to the sampling phase. It has only one sample available in one symbol period and is easily interfered by the channel, which does not allow the traditional symbol synchronization methods to be used directly. Another challenge is that the resource utilization for sampling data processing needs to be minimized due to the excessive consumption of the high data throughput in hardware resources. To solve these issues, we propose a symbol synchronization method based on the combination of software and hardware, which mainly includes two processes: Obtaining the initial phase by using Chirp signal and MOE criterion before communication; tracking the real-time phase using an on-line gradient table and frequency domain analysis of known data during communication. Both processes are proceeded with a phase adjustable clock. Through hardware verification, the sampling phase can be kept close to the optimal phase, thus ensuring the accuracy of the sampling data, and improving the system BER performance.

## 1. Introduction

E-band contains two continuous 5 GHz bandwidth from 71 to 86 GHz, which has been proved that has hundreds of Gbps transmission capability for point-to-point high-speed mm-Wave wireless communication systems [1]. As one of the most potential frequency bands for air-to-ground communication in the future 6G, a lot of work has proven its feasibility [2,3]. In pursuit of a higher wireless transmission rate, some research institutions or individuals have reported a realization rate of more than 100 Gbps [4]. The digital baseband system applied to ultra wide-band output is also toward higher rate research [5]. In order to achieve greater baseband bandwidth output, the most common way is to use frequency division with multiple narrow bands to synthesize a large broadband output [4,5]. This technology needs to reserve the protection band, leading to insufficient utilization of resources in the spectrum [6].

According to the Nyquist theorem, the required minimum sampling rate is twice of the signal bandwidth. Therefore, full Nyquist rate systems can make full use of the spectrum when converters are available [7]. Because the full Nyquist rate system with large bandwidth has high requirements for converter components, some researchers proposed a method with dual pulse-shaping to reduce the requirements for the device and realize the system [8]. Although, other technologies can achieve higher data rates, such as: Faster-than-Nyquist (FTN) technology [9] can reach a theoretical data rate of 25% higher than the full Nyquist rate. However, it is difficult to implement using the current commercial processors due to its high-complexity equalization [8]. Fortunately, it is demonstrated that the full Nyquist rate transmission can be achieved by the conventional low-complexity equalization method. Although full Nyquist rate transmission has many advantages, its sampling data will be changed unexpectedly with the offset of the sampling phase [10]. In order to realize the full Nyquist rate system, our work focuses on the symbol synchronization process of the system.

The purpose of symbol synchronization is to estimate and remove the fractional part of the unknown delay τ, which corresponds to the part of the error in [0,T) [11]. Therefore, this process is necessary for any communication system. According to the implementation process of symbol synchronization, there are three methods: a pure analog approach, a pure digital approach and a combined digital and analog approach where digital processing is used to correct the analog.

In pure analog and pure digital methods, the most typical one is the phase-locked loop (PLL). The analog phase-locked loop provides the locking basis through phase discrimination of the received signal [12,13]. Some use scene features, such as the relationship between space and electromagnetic wavelength to achieve synchronization, which is also a pure analog method [14]. A digital phase-locked loop is a phase acquisition method using signal characteristics [15]. The Gardner algorithm is also a method to obtain the timing phase by using signal characteristics for statistics [16,17]. Other methods of digital systems are based on data assistance, such as: data extraction of multiple sampling systems [18] or recovery of sampled data using known sequences [19,20]. For systems with fewer sampling dates, “early-late-gate” is a better method for data recovery [21,22]. A new purely digital method is: In the back-haul communication system with unbalanced in-phase and quadrature channel (IQ), the receiver obtains the phase difference by a digital method and feeds it back to the transmitter to realize the phase compensation by digital [23,24]. These pure digital methods are also generally applicable to analog digital mixed systems. In the combined digital and analog approach, the digital part uses the known sequence [25] or signal characteristics [26] to determine the current phase and feed it back to the hardware for adjustment.

Although there are many ways to achieve symbol synchronization, several changes should be taken into consideration when implementing a full Nyquist rate system with more than 100 Gbps data rate in E-band:

- Large data throughput makes the system not allow too many serial operations;
- The Symbol rate is equal to the sampling rate, which means that there is only one datum in a symbol period;
- No interpolation, no decimation, and no digital pre-shaping, the transmission pulse is ultimately shaped by the channel. So the arrival signal will be uncertain;
- Limited digital hardware resources, all kinds of processes need to be simplified;
- With high carrier frequency, it is difficult to obtain information related to phase discrimination on hardware.

When these characteristics need to be considered, the pure analog and the pure digital approaches cannot directly complete symbol synchronization. These methods, which extract phase information, also cannot be applied because of a lack of multiple data or iteration and data recovery algorithms with large hardware resource consumption. Without a digital pre-shaping and pre-compensation process, back-haul operation mode using IQ imbalance compensation is not feasible. So, in order to achieve symbol synchronization and reduce digital hardware resource consumption, we add a phase shifter to the sampling clock to achieve a hardware and software cooperative system. From the implementation process, our method can be classified as a combined digital and analog approach. But our method differs from training sequences [25] or signal features [26]. We use transmitter to generate Chirp signals. Simultaneous receiver estimates the offset value through the signal energy, and pre-adjust the sampling phase to clk board. This work ensures that accurate symbol synchronization before real-time data communication. Then, the training sequence is used to achieve phase tracking during the communication process.

The main contributions of this paper are as follows:

- Propose a method of quickly acquiring the initial phase before communication by using the characteristics of Chirp signal and maximum output energy (MOE) criterion. This method ensures that the system obtains the optimal phase immediately and rapidly after starting.
- Aiming at the fast change of the waveform caused by the channel, a decision method based on the frequency domain characteristics of the sampled signal is proposed. This decision ensures fast phase tracking of the system.
- A method of establishing a gradient table is proposed to solve the problem of slow phase offset. This process provides a long-term guarantee for sampling data accuracy.
- From the perspective of sampling and channel shaping, the difference between full Nyquist rate systems and conventional systems are analyzed.
- Numerical and hardware verifications show that the sampling phase is kept near the optimal phase, which ensures the stable output of effective data and thus improves the bit error rate (BER) performance of the system.

The rest of this paper is organized as follows. In Section 2, the architectures of the full Nyquist rate system and conventional systems are compared, and the problems of the full Nyquist rate system are analyzed from the perspective of sampling and channel shaping. In Section 3, the proposed method, implementation process and results are described. The test hardware, test process and joint test results are shown in Section 4 to evaluate the performance of the proposed method. Finally, conclusions are drawn in Section 5.

## 2. Nyquist Transmission Model

Compared to the conventional pulse-shaping systems, the transmission system, which with complementary Nyquist pulses has two differences: sampling and shaping process. In this section, we first contrast the conventional transmission systems and full Nyquist rate transmission system models. Next, we analyze the changes brought to the transmission system from these differences.

### 2.1. System Model

**Figure 1:**
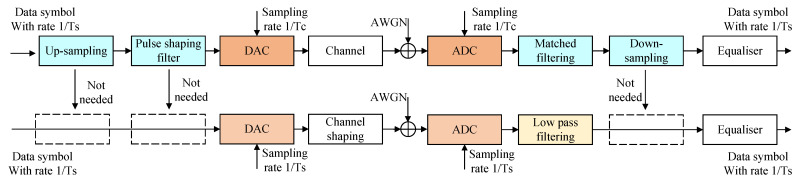
Conventional pulse shaping system (upper) and full Nyquist rate system (lower).

In order to make the converter work at the full Nyquist rate, the sampling rate needs to be equal to the symbol rate. Therefore, the digital pre-shaping process in conventional systems will not be implemented. Figure 1 shows the signal processing diagrams of the conventional pulse shaping system and full Nyquist rate system.

In the conventional pulse shaping system (Figure 1 upper), the process of up-sampling and down-sampling reduces the influence of the timing phase. Let s[m] denote the sequence of the transmitted signals. Sampling at the rate of 1/Tc, the output is:

(1) $$r_{c}\left(t\right)=\sqrt[{}]{E_{s}}\sum_{m=-\infty }^{\infty }s\left[m\right]g_{c}\left(t-mT_{c}\right)+v\left(t\right)$$

where $E_{s}$ denotes the average energy of the received signals after channel filtering, $g_{c}\left(t\right)$ is the channel impulse response and v(t) is additive white gaussian noise (AWGN).

Without up and down sampling and the pulse-shaping process, it has the advantage of obtaining a larger bandwidth and higher data rate for the full Nyquist rate system (Figure 1 lower), and it also brings higher sampling requirements. The influence of timing phase $\tau_{s}\;\left(\tau_{s}<T_{s}\right)$ needs to be considered. Sampling at $1/T_{s}$ rate:

(2) $$r_{s}\left(t\right)=\sqrt[{}]{E_{s}}\sum_{m=-\infty }^{\infty }s\left[m\right]g_{s}\left(t-mT_{s}-\tau_{s}\right)+v\left(t\right)$$

Compared with Equations (1) and (2), $g_{c}\left(t\right)$ is a certain waveform after pulse-shaping, but $g_{s}\left(t\right)$ is uncertain because the data has not undergone any digital processing. Meanwhile, the lack of up/down sampling makes $\tau_{s}$ need to be considered. The randomness of $\tau_{s}$ also makes it difficult to use the output data directly. Therefore, we need to study these variables further: $\tau_{s}$ and $g_{s}\left(t\right)$.

### 2.2. Nyquist Rate Sampling

For periodic or uniform sampling, a sequence of samples $\tilde{x}\left[n\right]$ is obtained from a continuous-time signal x(t) by taking values at an equally spaced point in time. The relationship between them is:

(3) $$\tilde{x}{\left[n\right]≜x\left(t\right)|}_{t=nT_{s}}=x\left(nT_{s}\right),-\infty <n<\infty$$

Supposing that the signal frequency domain representation before the analog to digital converter (ADC) is X(f), through frequency domain transformation of Equation (3), the received signal in the frequency domain after ADC is:

(4) $$\tilde{X}\left(e^{j2\pi fT_{s}}\right)=\frac{1}{T_{s}}\sum_{k=-\infty }^{\infty }X\left(f-k\frac{1}{T_{s}}\right)$$

*f* is the frequency variable, and *k* is the number of sample points in one symbol period.

Take the time delay $\tau_{s}$, which is introduced by adjusting the ADC clock phase into consideration. The received signal in the frequency domain after ADC can be expressed as:

(5) $$\tilde{X}\left(e^{j2\pi fT_{s}}\right)=\frac{1}{T_{s}}\sum_{k=-\infty }^{\infty }e^{-j2\pi \left(f-k\frac{1}{T_{s}}\right)\tau_{s}}X\left(f-k\frac{1}{T_{s}}\right)$$

Only one period processing $0\leq f\leq \frac{1}{T_{s}}$, k=0 and 1:

(6) $$\tilde{X}\left(e^{j2\pi fT_{s}}\right)=\frac{1}{T_{s}}\left[e^{-j2\pi f\tau_{s}}X\left(f\right)+e^{-j2\pi \left(f-\frac{1}{T_{s}}\right)\tau_{s}}X\left(f-\frac{1}{T_{s}}\right)\right]$$

Letting $X\left(f\right)=\left|X\left(f\right)\right|e^{j\varphi (f)}$, where φ(f) is the phase response of X(f):

(7) $$\tilde{X}\left(e^{j2\pi fT_{s}}\right)=\frac{1}{T_{s}}e^{-j2\pi f\tau_{s}}e^{j\varphi (f)}[|X\left(f\right)|+e^{j[2\pi \frac{\tau_{s}}{T_{s}}+\Delta \varphi \left(f\right)]}|X\left(f-\frac{1}{T_{s}}\right)\left|\right]$$

where $\Delta \varphi \left(f\right)=\varphi \left(f-\frac{1}{T_{s}}\right)-\varphi \left(f\right)$.

From Equation (7), we see the following situation exists:

(8) $$\tilde{X}\left(e^{j2\pi fT_{s}}\right)=0$$

when the conditions $|X\left(f\right)|=|X\left(f-\frac{1}{T_{s}}\right)|$ and $e^{j[2\pi \frac{\tau_{s}}{T_{s}}+\Delta \varphi \left(f\right)]}=-1$ are satisfied at the same time. This means that the sampled data under these conditions can’t be recovered, even if a good equalization algorithm is used.

Expand Equation (4) and retain k=0 and 1, which can easy to obtain an ideal sampling output:

(9) $${\tilde{X}}_{ideal}\left(e^{j2\pi fT_{s}}\right)=\frac{1}{T_{s}}[|X\left(f\right)|+|X\left(f-\frac{1}{T_{s}}\right)\left|\right]$$

Note: Ideally, $e^{j\varphi (f)}$ can be ignored.

Comparing Equations (7) and (9), we can get the amplitude–frequency characteristics are related to the sampling phase $\tau_{s}$ and different frequencies show different amplitudes.

Summary I:

- Spectral null data may exist in the sampling output;
- The data will not only have amplitude changes, but also show frequency selection characteristics.

### 2.3. Channel Shaping

**Figure 2:**
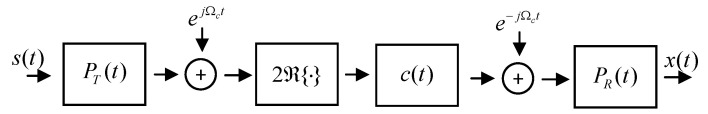
Block diagram of the channel model in a digital communication system.

Since there is no digital pre-shaping process, the transmit beam will be shaped by the channel completely. Figure 2 presents the block diagram of a digital communication system.

In Figure 2, $P_{T}\left(t\right)$ and $P_{R}\left(t\right)$ as analog low-pass filters, the block *ℜ* takes the real part of the carrier band processing result, c(t) is channel, $e^{j\Omega_{c}t}$ and $e^{-j\Omega_{c}t}$ represents up-conversion and down-conversion, respectively, $\Omega_{c}$ is the carrier frequency. We expected x(t)=s(t), that is, the output waveform is identical to the input waveform. However, in the full Nyquist rate system, the quadrature amplitude modulation (QAM) signal will be directly sent to the digital to analog converter (DAC). The signal has a high-frequency band and poor continuity. These features are very unfavorable for long-distance wireless transmission, and the output waveform will be changed by the carrier conversion and channel.

From the conditions of without inter symbol interference (ISI) transmission, the waveform must be sinc function. For a complex baseband signal, when the bandwidth *B* is sufficiently smaller than the carrier frequency $F_{c}$: $B\ll f_{c}$, the down-conversion output can be expressed as:

(10) $$x\left(t\right)=2Bsinc\left(tB\right)*(x_{p}\left(t\right)e^{-j2\pi f_{c}t})$$

$x_{p}\left(t\right)$ is the passband output signal. Ideally, there is

(11) $$x_{p}\left(t\right)=ℜ\left[s\left(t\right)e^{j2\pi f_{c}t}\right]$$

Equation (11) describes the up-conversion process. From Equation (10), we can get: for E-band transmission with carrier frequency of 73.5 GHz and bandwidth of 5 GHz, without ISI conditions are met. However, due to the existence of c(t), the $x_{p}\left(t\right)$ in Equations (10) and (11) are not the same. Therefore, we studied ideal channel and complex channel shaping and their BER performance.

**Figure 3:**
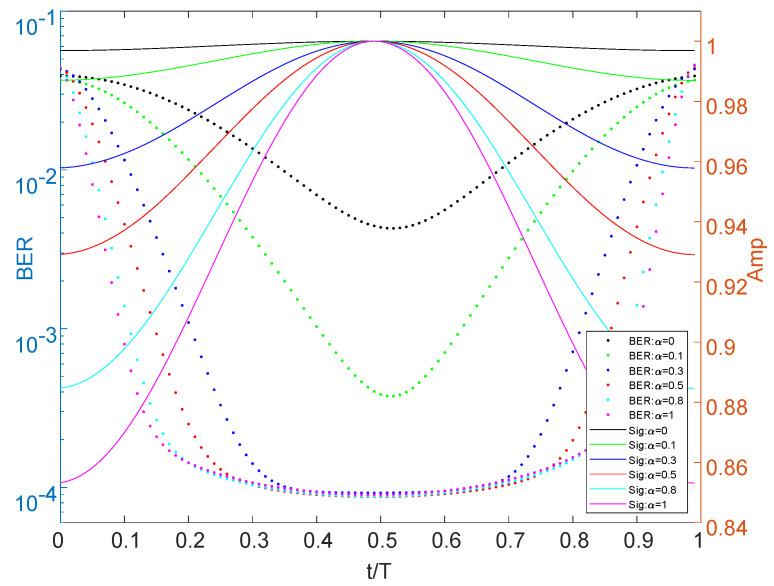
Signal and BER for ideal channel.

**Figure 4:**
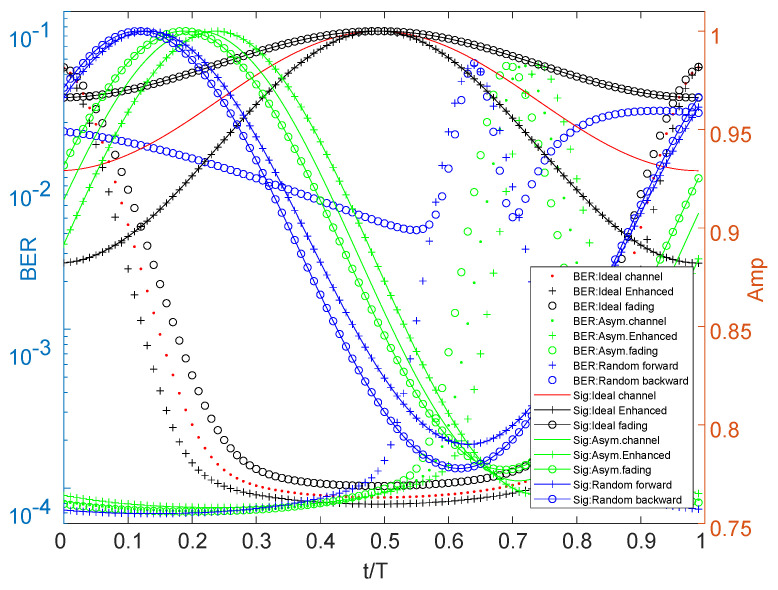
Signal and BER for complex channel.

In order to generate full Nyquist rate pulse-shaped signals in the simulation, the QAM symbols are sent directly to an RC filter for performing analog shaping, which is implemented with a Sinc impulse response.The ideal channel impulse response function is given by: g(t)=δ(t)+0.5δ(t−0.5). Other models vary from this relationship. The simulation results of three models are shown in Figure 3 and Figure 4. All models adopt a minimum mean squared error (MMSE) equilibrium method.

Figure 3 compares different roll-off coefficients, which are directly reflected in the change of signal amplitude and BER. From Figure 4, the enhanced or fading channel also brings out the amplitude and BER change. The peak value of the signal will shift with the non-ideal channel, and even have a poor BER for some less common channels.

Summary II:

- In E-band wireless communication, full Nyquist rate transmission architecture is used to meet the requirements without ISI;
- Shaped by an analog channel, unknown changes such as amplitude and phase may occur.

From summary I and II, the basic transmission conditions are met, but the changes in amplitude and phase caused by analog channel shaping and sampling delay bring out poor data are unpredictable, which also increases the difficulty of system data recovery. Although the channel cannot be changed, it can still combine its characteristics to achieve accurate data acquisition from the perspective of sampling phase control.

## 3. Methods

From the system model, it can be seen that the sampling clock with phase modulation function is necessary to realize the system. By adjusting the sampling time delay, we can avoid Equation (8), so spectral null will not happen. Additionally, we can keep the sampling working around the signal energy peak, in order to obtain optimal data.

### 3.1. Maximum Output Energy (MOE) Criterion

In order to achieve accurate control, precise estimation is required first. Maximum likelihood estimation is the most common method, such as the early–late gate approach, exploit filter banks and so on. For E-band communication with large data throughput, resource consumption must be considered. Therefore, the above methods are costly. From the perspective of the channel shaping effect, an approach based on a cost function known as the maximum output energy (MOE) criterion may be the best choice.

When the sampling delay parameter τ is introduced, it is necessary to study the signal changes during a single symbol period, so the transmitted signal can be identified as s[m]=1. Sampling from Equation (2) by $nT_{s}+\tau$, the output energy is:

(12) $$J_{MOE}\left(\tau \right)=E[|r_{s}\left(nT_{s}+\tau \right){|}^{2}]=E_{s}\sum_{m=-\infty }^{\infty }{\left|g_{s}\left(mT_{s}+\tau -\tau_{s}\right)\right|}^{2}+N_{0}$$

$N_{0}$ is noise energy.

The rationale behind the MOE criterion is that:

(13) $$E[|r_{s}\left(nT_{s}+\tau \right){|}^{2}]\leq E_{s}{\left|g_{s}\left(0\right)\right|}^{2}+N_{0}$$

This equation holds for sinc type functions, and can be verified from channel shaping results (Figure 3).

Based on this criterion, the optimal initial sampling phase can be quickly found before communication is established, and real-time phase tracking can be carried out during communication.

### 3.2. Initial Phase Acquisition

MOE proposes a method to find ideal data from the signal energy. Our work is obtaining the corresponding phase value of the data, so we need to transform the parameters from Equation (13).

First, we should clarify the relationship between τ and $g_{s}$:

(14) $$g_{s}\left(\tau_{optimal}\right)=g_{s}\left(0\right)$$

That is, the optimal sampling phase corresponds to the maximum amplitude, and the following relations are also satisfied:

(15) $$g_{s}\left(0\right)=max\left(g_{s}\left(t\right)\right)$$

Here, our task is to find the optimal τ; therefore, the work is converted to:

(16) $$\hat{\tau }=argmaxJ_{MOE}\left(\tau \right)$$

This process needs to be completed within a $T_{s}$ period, so the tau value range is:

(17) $$\tau \in [0,T_{s})$$

Second, we need to solve the variables involved one by one. According to the actual situation, $g_{s}\left(t\right)$ is the channel, and it belongs to an unknown quantity. Additionally, $g_{s}\left(0\right)$ is unknown. However, a known waveform can be used to reduce the channel impact, and the phase can be acquired before the communication is established. This practice needs to be completed under specific conditions:

- The average power change of the received signal is maintained in a constant range in a short time;
- The sampling clock frequency offset of hardware changes very slowly relative to the symbol rate;
- The channel needs to remain fixed during this process.

Next, a reliable waveform needs to be selected. The waveform should have periodicity, be able to output wide-band signals, and have good amplitude–frequency characteristics. Linear frequency modulation signal Chirp is used as the periodic signal, which meets all needs [27]. The plural form is:

(18) $$x_{Chirp}\left(t\right)=e^{j\pi Bt^{2}/\tau_{c}}=\sin\left(\pi Bt^{2}/\tau_{c}\right)+j\cos\left(\pi Bt^{2}/\tau_{c}\right)$$

**Figure 5:**
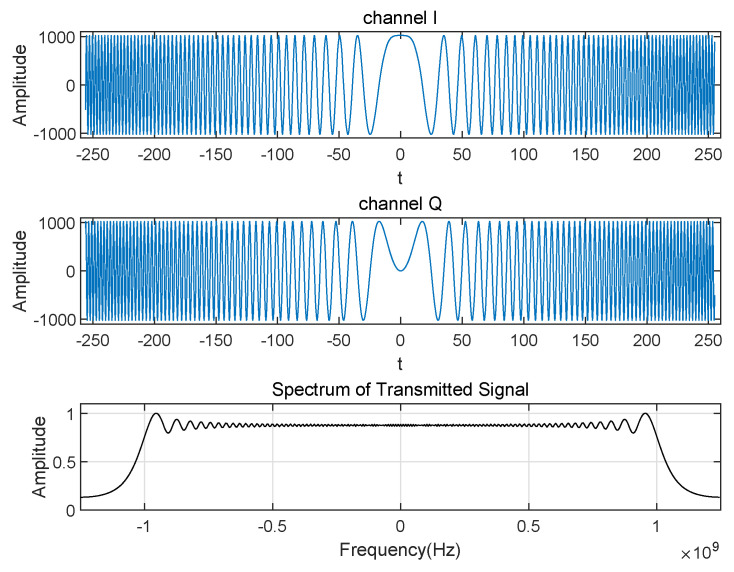
Chirp signal and spectrum.

Another advantage of Chirp signal is that it can be divided into real part and imaginary part, each part has same amplitude, same frequency and same bandwidth. Use the orthogonality between them, when the signal is sent to the IQ channels, respectively, the receiver can easily obtain the order of the two channels. Figure 5 shows 512 point Chirp signal and spectrum.

**Figure 6:**
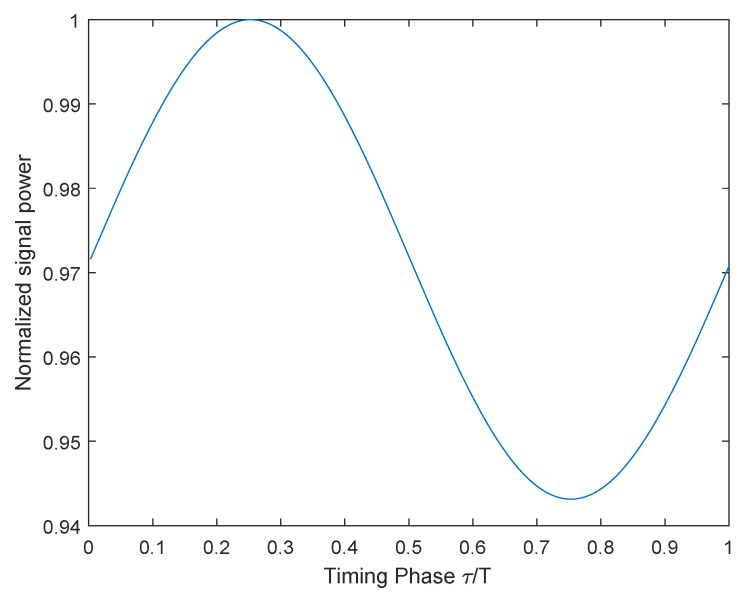
Signal energy of one phase shifting period.

Finally, we can obtain the optimal phase. This process cannot obtain $g_{s}$ directly, but the total energy of the sequence can be obtained. By cyclically adjusting the phase, we can access a group of energy values. Then, the maximum value can be found from this group of data, which corresponds to g(0), and it also corresponds to the optimal phase $\tau_{optimal}$. So, the Chirp signal is sent continuously and periodically, and the sampling phase of the hardware is adjusted simultaneously. The receiver calculates the energy of the data in the same periods and finds the sampling phase corresponding to the maximum energy. The energy change of Chirp in one phase shift period is given in Figure 6.

After angle calculation, the optimal phase in Figure 6 is $91^{\circ }/360^{\circ }$. Therefore, adjust the sampling clock to shift the phase by $91^{\circ }$.

Another reason we choose Chirp is that this process can also intuitively obtain the imbalance between IQ channels. When the maximum energy values of the two are different, one of them can be used as a reference, and the amplitude balance can be achieved by pre-compensating the other. By adjusting the sampling clock of the two channels, respectively, the phase balance between them can be achieved.

### 3.3. Real-Time Phase Tracking

Figure 4 also draws a conclusion that: when the channel changes, it is very easy to change the optimal phase. In data communication, if it cannot be adjusted in time, a bad BER will occur, even leading to transmission interruption. Due to the possibility of Equation (8), the data which are sampled at “spectral null” cannot be output accurately by using data recovery methods such as equalization or “early-late-gate”. This process can be expressed as fast channel change. There is also a slow offset that occurs slowly over time. Two different effects require different decision methods. Next, we will discuss the implementation method in combination with multiple conditions such as signal and sampling.

In the previous process, Chirp was used to obtain $g_{s}\left(t\right)$ indirectly. However, in this process, with Chirp as additional data, if they are added, the transmitted valid data will be reduced. Thus, we need to reselect the fixed data or sequence to replace $g_{s}\left(t\right)$ and g(0). In the frame structure, the sequence and pilots are set to known data, it is usually used as the main way to obtain the equilibrium coefficient or various parameters. We can also use them to obtain the decision basis of phase tracking.

**Figure 7:**
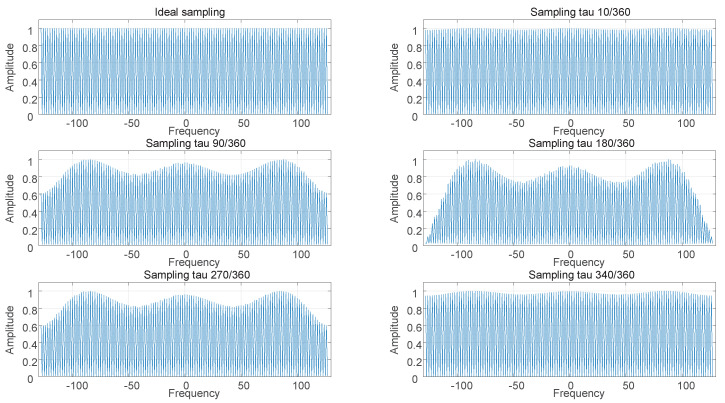
Frequency domain analysis of sequences in different $\tau_{s}$.

During data communication, the preamble and pilots will be changed by the carrier frequency offset, channel fading, and so on. Because these may cause changes in signal energy, it is difficult to achieve accurate judgment by MOE alone. In Equation (7) or summary I, when the sampling phase shift occurs, the output results reflect the frequency selection characteristics. Back to Equation (7), the common coefficient is $\frac{1}{T_{s}}e^{-j2\pi f\tau_{s}}e^{j\varphi (f)}$. Although $\tau_{s}$ is unknown, it can still be considered as fixed at a certain time. At this time, the coefficient part is the expression covering *f*. This conclusion is still valid for the coefficients $e^{j[2\pi \frac{\tau_{s}}{T_{s}}+\Delta \varphi \left(f\right)]}$ of the second part of the polynomial. Therefore, we substitute different $\tau_{s}$ into the simulation to obtain the amplitude–frequency characteristics after sampling. Figure 7 shows frequency domain analysis of sequences in different $\tau_{s}$.

**Figure 8:**
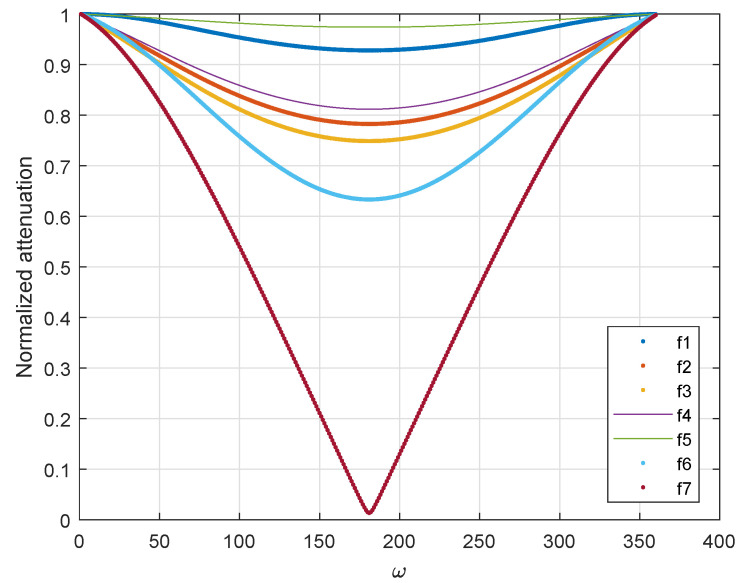
Attenuation of different frequencies in different phases.

Since the output is not a linear function of *f*, different frequencies exhibit different degrees of attenuation. By comparing different sampling phases in Figure 7: Some frequency bands have almost no attenuation, but some frequency bands directly attenuate to a lower value when the phase shift is large. The attenuation curves of different frequencies at different phases are shown in Figure 8. The relationship of each frequency is: f1<f2<f3<f4<f5<f6<f7.

It can be seen from the above figure that:

- Different frequencies have different attenuation amplitudes;
- Some high frequencies reflect slow attenuation, but the highest frequency is sensitive to phase change;
- The attenuation curve does not have linearization;
- The attenuation variation is slight around a small phase change interval;

By tracking the frequency with large attenuation, we can quickly know whether there is a fast channel change. For a small phase offset, data compensation can be realized through equalization, but for large offset, it is necessary to estimate to obtain an accurate value reasonably. Therefore, we still need to take some measures.

Unlike the initial phase acquisition process, only g(0) needs to be found. In this process, there will be two possibilities for the sampling value when the phase is shifted. It is difficult to determine whether it is left or right by simply comparing with g(0). Therefore, our work is mainly to obtain the direction of the offset and the approximate distance of the offset.

Back to Figure 6, the normalized energy relationship is given, which not only reflects the changing trend, but can also obtain the offset distance through comparison. Using this principle, a gradient table based on Figure 6 can be established. Through real-time energy comparison, the problem of offset and distance can be solved.

Assuming that the normalized gradient table is α(τ) and the average energy of the previous frames are $\bar{E}$, the complete gradient network table is:

(19) $$\rho \left(\tau \right)=\bar{E}*\alpha \left(\tau \right)$$

The normalized gradient table α(τ) in (19) establishes the relationship between clock phase and signal energy. Its actual plotting curve is similar to (Figure 6). It can be seen that one clock phase only corresponds to one normalized energy value, while one normalized energy corresponds to multiple phases. In order to find out the relationship from energy to phase, we need to find out the energy changing trend from the energy change curve. By combining real-time calculated energy and changing trend, we can obtain the unique mapping relationship from energy to phase.

Substitute the current energy *E* into ρ(τ) for numerical comparison, and return τ. The offset distance is obtained. By comparing whether the energy of the previous frames increases or decreases, we can get the amplitude changing trend and phase offset direction. This process can also provide a reference for slow offset. Then the τ can be sent to the hardware for tracking adjustment after smooth filtering.

**Figure 9:**
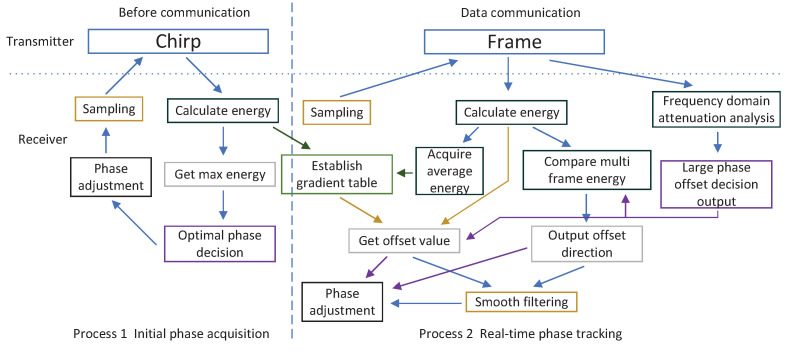
The main procedures of phase acquisition and tracking processes.

The main procedures of the two processes can be shown in Figure 9:

The process in Figure 9 can be summarized as:

- Before communication, the optimal phase is obtained through Chirp, and the basic data of the table is provided for the follow-up steps;
- In the communication process, frame sequence is used to complete time domain energy calculation and frequency domain phase determination;
- The average energy will be obtained to improve the gradient table;
- Multi-frame energy is used to calculate offset direction;
- Slow offset needs to be filtered smoothly, while fast channel transformation will be sent to adjust directly.

The method does not require complicated signal processing and excessive resources. Some processes can be further adjusted to minimize hardware resource consumption. For example, process 1 can be performed with the signal generators or coprocessors. After the completion of process 1, only the normalized energy table needs to be sent to the digital signal processor. Only the frequency domain decision and energy comparison steps need to be added, because these blocks, such as frequency domain and energy calculation, have been implemented in the digital signal processing. The offset value calculation, change trend analysis and other processes can still be handed over to the signal generators or coprocessors. Therefore, this method can appropriately adjust the resource consumption according to the hardware.

## 4. Hardware Verifications and Results

**Figure 10:**
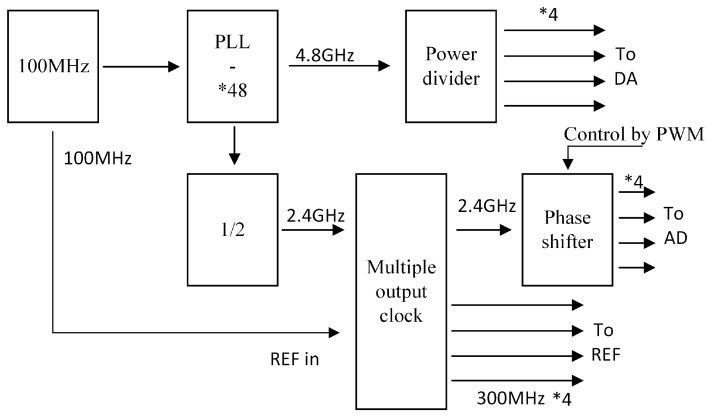
Clock system with 4-way synchronous output with phase adjustable function.

According to the above discussion, a clock system with a phase adjustable function is necessary. In order to suppress interference between adjacent frequency bands, the system uses 4.8 GHz instead of 5 GHz. Figure 10 shows the block diagram of a four-channel synchronous output clock system with phase adjustment function.

**Figure 11:**
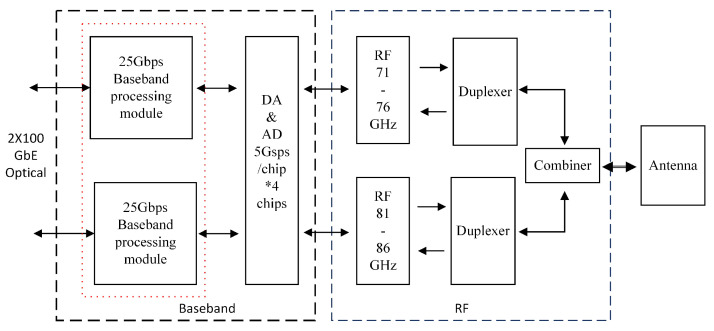
Block diagram of E-band wireless transceiver platform.

To realize phase control, the delay of phase shifter must be considered. Although the digital phase shifter is simple to control, its response time is long. The analog phase shifter has complex control but fast response. Through the analysis of system requirements, we choose an analog phase shifter based on pulse width modulation (PWM) control. Another parameter of the phase shifter is precision. It is undeniable that the higher the accuracy, the better the implementation effect, but the complexity of the algorithm also increases, and the cycle time of the initial phase acquisition is also longer. Since the equalizer can compensate the data to a certain extent, we choose a phase shifter with a minimum adjustment accuracy of 1.4 degrees, which fully meets the tracking accuracy and reasonable resource utilization. The maximum gradient of hardware adjustment is 257, and the minimum phase is 0.0039$T_{s}$. The clock system is applied to the E-band millimetre wave wireless transceiver platform of Figure 11.

The platform can transmit or receive 71–76 GHz and 81–86 GHz at the same time. The baseband includes 4 chips of DAC and ADC, which can output or input 2 channels of orthogonal IQ signals. The data of the two frequency bands can be processed jointly or divided into independent data streams.

**Figure 12:**
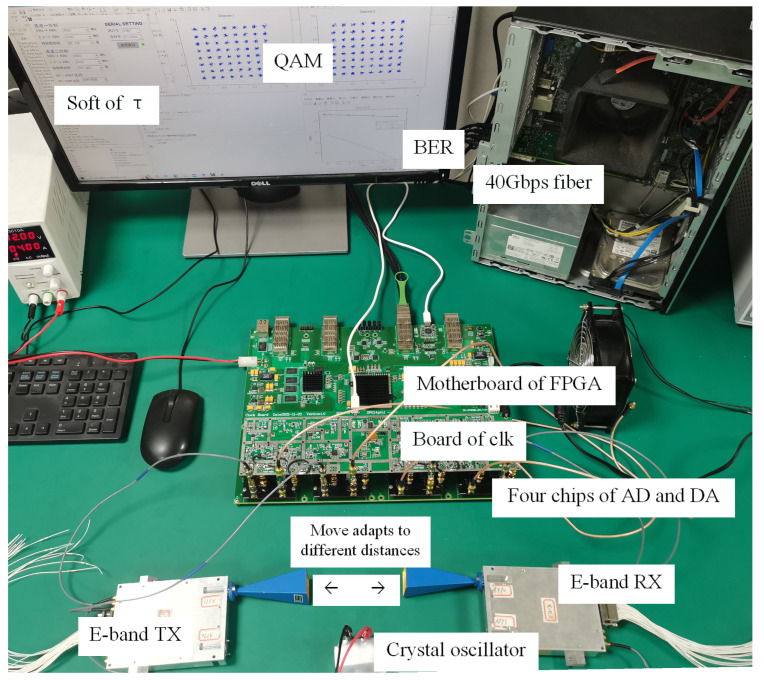
The test scene of simulating phase change by moving the antenna and adjusting the power.

The phase acquisition and adjustment algorithm are applied to the core processor. Because the phase of signal arrival cannot be directly obtained, using the relationship between the electromagnetic wavelength and the path, we change the arrival phase by moving the transceiver antenna for testing. Moving the antenna adapts to different distances between the transmitter and receiver can appropriately change the phase of signal arrival, and experimental tests can be conducted through this method. 16 QAM and 64 QAM were tested many times, and the relevant data were obtained. Figure 12 shows the test scene of simulating phase change by moving the antenna and adjusting the transmit and receive power.

The data is exchanged between the computer and the motherboard of FPGA through 40 Gbps optical fiber. The computer outputs the generation of data, calculates the BER and controls the clock. The motherboard of FPGA realizes data processing, signal conversion and output energy control. Wireless signals are transmitted by E-band RF front-end and antennas. The external crystal oscillator provides accurate carrier frequency signals.

**Figure 13:**
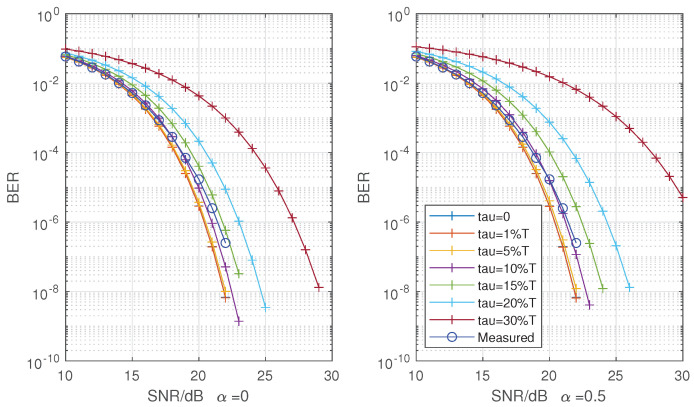
Measured BER of 16 QAM and theoretical at roll-off α = 0 (**left**) and α = 0.5 (**right**) with RC.

**Figure 14:**
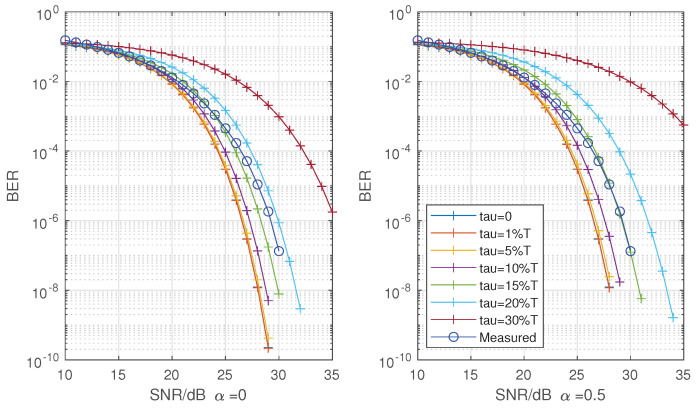
Measured BER of 64 QAM and theoretical at roll-off α = 0 (**left**) and α = 0.5 (**right**) with RC.

Due to the distance of the transceiver antenna, hardware loss, the uncertainty of initial phase difference of transceiver clock after start-up and other factors, the value of the initial phase adjustment does not have regularity. At the same time, the probability of phase offset is low during the operation of the system, and the process of real-time tracking and adjustment is sudden. Therefore, it is difficult to show the statistical relationship between phase change and adjustment in hardware operation, so the implementation effect is shown from the perspective of BER. Figure 13 and Figure 14 show the measured BER of 16 QAM and 64 QAM and theoretical BER at roll-off 0 and 0.5 with root raised cosines (RC) filter.

The measured values in Figure 13 and Figure 14 are a statistical result of multiple measurements made by the hardware under the condition of establishing reliable communication. The result may be affected by the number of measurement symbols, frequency selection characteristics of hardware, actual channel, clock precision, algorithm and other factors. According to theory, the full Nyquist rate pulse is a function of Sinc. In fact, it may be the secondary or multiple modulation function of Sinc. This problem makes it impossible to give an ideal reference condition qualitatively, and affects the accuracy of the “gradient table” in the algorithm. The algorithm constrains the maximum phase offset of 0.0507$T_{s}$, that is, the minimum range of smooth filter adjustment is 12$\tau_{min}$. The measured results calculated count the error code to $10^{-6}$. Compared with the BER performance loss, 16 QAM only 1 dB and 64 QAM loses 3 dB. Considering the influence of sampling phase offset, when the sampling delay exceeds 30%$T_{s}$, the system without phase adjustment will have poor performance of BER. The method proposed in this paper can ensure that it is within an acceptable range. From different roll-off factors, the adjusted bit error offset is within 10–15%$T_{s}$ of RC theoretical phase offset at 16 QAM and 15–20%$T_{s}$ at 64 QAM. Although some loss of performance cannot be avoided through the method or unknown channel shaping, but the sampling can be maintained in a low offset range of optimal sampling phase to ensure the stable operation of the system.

## 5. Conclusions

In this paper, we propose a hardware and software cooperation method to realize symbol synchronization in full Nyquist rate systems. Our proposed method does not require complicated digital signal processing and occupy too many resources. It also can provide flexible resource configuration, and choose to use direct processing or co-processing according to the existing hardware. With this method, the sampled data can be accurately obtained in the process of data transmission, which enables the full Nyquist rate transmission. When the converter is restricted, the full Nyquist rate system implemented by our proposed method can achieve twice the bandwidth and data rate improvement compared with conventional pulse-shaping systems. In future work, we will continue to explore larger bandwidth systems, e.g., FTN systems and Tera-Hertz (THz) systems, to achieve accurate data acquisition. Meanwhile, for the current system, we explore how to combine our proposed method with IQ compensation, and further improve the SNR and BER performance by building a back-haul communication. 

## Data Availability

Not applicable.

## Abbreviations

The following abbreviations are used in this manuscript:

AWGN	Additive White Gaussian Noise
ADC	Analog to Digital Converter
QAM	Quadrature amplitude modulation
DAC	Digital to Analog Converter
ISI	Inter symbol interference
RC	Root raised cosines
MMSE	Minimum mean squared error
BER	Bit error rate
MOE	Maximum output energy
PWM	Pulse width modulation
PLL	Phase-locked loop
FTN	Faster than Nyquist
MIMO	Multiple-in multiple-out
THz	Tera-Hertz

## References

[1] Nie S., MacCartney G., Sun S., Rappaport T. 28 GHz and 73 GHz signal outage study for millimeter wave cellular and backhaul communications. Proceedings of the 2014 IEEE International Conference on Communications (ICC).

[2] Hamza A., Deogun J., Alexander D. (2019). Classification Framework for Free Space Optical Communication Links and Systems. IEEE Commun. Surv. Tutor..

[3] Huang X., Zhang J., Liu R., Guo Y., Hanzo L. (2019). Airplane-Aided Integrated Networking for 6G Wireless: Will It Work?. IEEE Veh. Technol. Mag..

[4] Czegledi C. Demonstrating 139 Gbps and 55.6 bps/Hz Spectrum Efficiency Using 8 × 8 MIMO over a 1.5-km Link at 73.5 GHz. Proceedings of the 2020 IEEE/MTT-S International Microwave Symposium.

[5] Zhang T., Zhang H., Huang X., Suzuki H., Pathikulangara J., Smart K., Du J., Guo J. (2022). A 245 GHz Real-Time Wideband Wireless Communication Link with 30 Gbps Data Rate. Photonics.

[6] Yousefi M., Yangzhang X. (2020). Linear and Nonlinear Frequency-Division Multiplexing. IEEE Trans. Inf. Theory.

[7] Lu Y., Lv S., Wang X. (2019). Adaptive Sub-Nyquist Spectrum Sensing for Ultra-Wideband Communication Systems. Symmetry.

[8] Li H., Huang X., Zhang J., Guo Y. (2022). Dual pulse shaping transmission with sinc function based complementary Nyquist pulses. IET Commun..

[9] Li S., Yuan W., Yuan J., Bai B., Wing D., Hanzo L. (2020). Time-Domain vs. Frequency-Domain Equalization for FTN Signaling. IEEE Trans. Veh. Technol..

[10] Clark K., Liu Z. (2022). Modeling the Performance of the Clock Phase Caching Approach to Clock and Data Recovery. J. Light. Technol..

[11] Lu C., Zhigang C., Zhi J. (2022). Maximization-Based Simultaneous Localization and Mapping for Millimeter-Wave Communication Systems. Sensors.

[12] Liu B., Guo X., Kong W., Liu T., Dong R., Zhang S. (2022). Stabilized Time Transfer via a 1000-km Optical Fiber Link Using High-Precision Delay Compensation System. Photonics.

[13] Yang C., Liu X., Jin J., Guo Y., Zhou J. (2022). A Fast-Settling Phase-Locked Loop Utilizing Cycle-Slipping-Elimination PFDCP. IEEE Trans. Circuits Syst. II Express Briefs.

[14] Wang K., Liu Y., Hong Z., Lin M. (2021). A Novel Timing Synchronization Method for DCO-OFDM-Based VLC Systems. IEEE Photonics J..

[15] Li S., Wang S., Zhao C., Cui X., Liu J. (2022). A Non-Data-Aided Feedforward Timing Estimator Based on Multiple Cyclic Correlations for Short-Term Burst Signals. IEEE Commun. Lett..

[16] Hao X., Lin C., Wu Q. (2020). A Parallel Timing Synchronization Structure in Real-Time High Transmission Capacity Wireless Communication Systems. Electronics.

[17] Gu Y., Cui S., Ke C., Zhou K., Liu D. (2019). All-Digital Timing Recovery for Free Space Optical Communication Signals with a Large Dynamic Range and Low OSNR. IEEE Photonics J..

[18] Pan P., Wang H., Shen L., Lu C. (2019). Equivalence of Joint ML-Decoding and Separate MMSE-ML Decoding for Training-Based MIMO Systems. IEEE Access.

[19] Hu W., Wang Z., Mei R., Lin M. (2021). An Efficient Carrier Synchronization Scheme for Demodulation Systems. Electronics.

[20] Shen Y., Shi X., Zhao S., Wang Y. (2022). An Improved Phase Deviation Discriminator for Carrier Synchronization of APSK Signal in Satellite-to-Ground Communication Systems. Electronics.

[21] Yadav K., Hsieh P., Carusone A. (2022). Loop Dynamics Analysis of PAM-4 Mueller–Muller Clock and Data Recovery System. IEEE Open J. Circuits Syst..

[22] Yuan Y., Luo Y., Zhang Q., Zhang C. (2022). TFR Recovery From Incomplete Micro-Doppler Signal via AL-ADMM-Net. IEEE Access.

[23] Abdallah S., Salameh A., Saad M. (2021). Joint Channel, Carrier Frequency Offset and I/Q Imbalance Estimation in Ambient Backscatter Communication Systems. IEEE Commun. Lett..

[24] Lu L., Ma X., Liang Y., Liu Z., Fan X., Li L. (2022). A 60-GHz Hybrid FMCW-Doppler Radar for Vibration Detection With a Robust I/Q Calibration Method. IEEE Sens. J..

[25] Yang G., Zhou F., Qiao G., Zhao Y., Liu Y., Lu Y., He Y. (2021). Optimized Doppler Estimation and Symbol Synchronization for Mobile M-ary Spread Spectrum Underwater Acoustic Communication. J. Mar. Sci. Eng..

[26] Wang D., Guo R., Liu L., Yuan H., Li X., Pan J., Tang C. (2022). A Method of Whole-Network Adjustment for Clock Offset Based on Satellite-Ground and Inter-Satellite Link Observations. Remote Sens..

[27] Berggren F., Popović B. (2022). Joint Radar and Communications With Multicarrier Chirp-Based Waveform. IEEE Open J. Commun. Soc..

